# Development of Seven New dPCR Animal Species Assays and a Reference Material to Support Quantitative Ratio Measurements of Food and Feed Products

**DOI:** 10.3390/foods12203839

**Published:** 2023-10-20

**Authors:** Kate R. Griffiths, Jacob L. H. McLaughlin, Felicity Hall, Lina Partis, Samuel C. Hansen, Rachel Tulloch, Daniel G. Burke

**Affiliations:** Bioanalysis Section, National Measurement Institute, Lindfield, Sydney, NSW 2070, Australia

**Keywords:** duplex digital PCR, dPCR, animal species, synthetic reference materials, ratio measurements, meat and bone meal, petfood, food fraud

## Abstract

Laboratory testing methods to confirm the identity of meat products and eliminate food fraud regularly rely on PCR amplification of extracted DNA, with most published assays detecting mitochondrial sequences, providing sensitive presence/absence results. By targeting single-copy nuclear targets instead, relative quantification measurements are achievable, providing additional information on the proportions of meat species detected. In this Methods paper, new assays for horse, donkey, duck, kangaroo, camel, water buffalo and crocodile have been developed to expand the range of species that can be quantified, and a previously published reference assay targeting the myostatin gene has been modified to include marsupials and reptiles. The accuracy of this ratio measurement approach was demonstrated using dPCR with mixtures of meat DNA down to 0.1%. However, the limit of detection (LOD) of this approach is not just determined by the assay targets, but by the samples themselves, with food or feed ingredients and processing impacting the DNA yield and integrity. In routine testing settings, the myostatin assay can provide multiple quality control roles, including monitoring the yield and purity of extracted DNA, identifying the presence of additional meats not detected by the suite of species-specific assays and potentially estimating a sample-specific LOD based on measured copy numbers of the myostatin target. In addition to the myostatin positive control assay, a synthetic DNA reference material (RM) has been designed, containing PCR targets for beef, pork, sheep, chicken, goat, kangaroo, horse, water buffalo and myostatin, to be used as a positive template control. The availability of standardised measurement methods and associated RMs significantly improves the reliability, comparability and transparency of laboratory testing, leading to greater confidence in results.

## 1. Introduction

The risks associated with the adulteration of meat products have far-reaching impacts beyond incorrect labelling, with global costs associated with food fraud estimated to be AUS $40–50 billion a year, impacting safety, health and trust [1]. In the early 2000s, when the outbreak of Bovine spongiform encephalopathy (BSE) or “Mad Cow Disease” was linked to rendered ruminant meat meal in cattle feed [2], the World Health Organisation (WHO) placed an international ban on this practice to reduce the spread of disease. Meat and bone meal (MBM) from different animal species had to be separated, with ongoing testing to ensure the segregation was effective. Rendered MBM from different species also has different market values, with the substitution of a premium MBM with a cheaper species difficult to distinguish by appearance or smell alone; consequently, adulteration can be easily concealed. In the years following the MBM ban, other drivers and exclusions have impacted the meat rendering industry: new disease outbreaks, cultural or religious requirements [3] and the European horse meat scandal of 2013 [4,5]. MBM is a commonly used ingredient in the pet food industry and this market is driven by the need to support dietary and allergy requirements or consumer choice, which rely on the production of different recipes.

At present, there are no industry-endorsed methods or between-lab consistency for measuring the proportions of different species in MBM. Unsatisfactory results can potentially lead to whole shipments of MBM being rejected, trade embargos being established and millions of dollars in losses, such as the 2018 suspension of Australian beef MBM into Indonesia, costing AUS $100 million annually (personal communication from Australian Renderers Association (ARA) executive members). When there is no suitable or standardised testing method, exporters are unable to confirm the validity of test results from overseas laboratories.

The majority of MBM testing uses the DNA amplification method Polymerase Chain Reaction (PCR), as it can detect even traces of fragmented DNA that survived the high temperatures and pressure from the rendering process. This method is equally suitable for the analysis of most meat-based food products for determining meat identity and food fraud [6,7,8].

As the initial driver for species segregation of MBM was disease control, PCR assay designs focused on method sensitivity, allowing even traces of fragmented DNA to be detected. These assays usually target mitochondrial genome sequences that are present in hundreds of copies per cell to improve the LOD [2,8,9,10,11,12,13,14]. If an unexpected species is detected, its presence may need to be quantified to determine if this is a trace contamination or potential adulteration. Whilst mitochondrial targets are ideal for presence/absence testing when method sensitivity is the priority, they are not suitable for quantification [6,15]. The number of mitochondria per cell varies significantly, between 300–4000 per cell, depending on the function of the tissue and its energy needs [12,15]. This means the copy number of mitochondrial DNA targets per cell also varies significantly between tissue types, making relative quantification highly inaccurate. Most meat processed for human consumption is muscle tissue, with a somewhat consistent range of mitochondrial copies per cell. MBM, however, contains animal parts not for human consumption [16], including offal, bone, hooves and feathers. The variation in copies of mitochondria per cell is significantly increased when dealing with this undefined variety of tissue types.

For measuring the relative proportions of different species present in meat products, “single-copy per haploid nuclear genome” genes are more appropriate as there are two copies of the target in each eukaryote cell [6,15,17,18], with the exception of the few multi-nucleated cell types. This species-specific measurement approach was developed by Köppel and his colleagues at the Official Food Control Authority of Canton Zürich in Switzerland, allowing accurate measurements of cell ratios in meat products [19,20]. The measurement uncertainty of these ratio measurements, when comparing quantitative PCR (qPCR) to digital PCR (dPCR), showed better measurement accuracy and precision with dPCR [21,22]. Digital PCR is a more recent iteration of the DNA amplification process, with the significant advantage over qPCR being that no calibrant is required to produce quantitative data, and results are less impacted by inhibition [23]. The DNA extracted from the test sample is mixed with the PCR assay reagents and then divided into several thousand partitions before amplification. This partitioning can occur in a physical chip format, in the case of the Qiagen QIAcuity platform, or by the production of an emulsion containing thousands of spherical droplets, each forming individual PCRs, as in the Bio-Rad QX200 platform. The DNA template is diluted prior to mixing so that not all partitions have a copy of the PCR target. Those with at least one copy of the template will be positive at the end of the amplification and those with no template will be negative, hence the binary digital nature of the output. PCR success is monitored using intercalating fluorescent dyes or Taqman^®^ probes in the same way as qPCR, but quantification is measured by calculating the number of positive droplets or partitions relative to the total number counted and applying Poisson statistics to calculate the probability of a positive droplet containing more than one copy of the template at the start of the PCR [23]. The performance of this measurement approach for meat species ratios was successfully demonstrated in a ring trial, using cooked sausage meat made from mixtures of beef, pork, lamb, chicken, turkey and horse [18].

Following this trial, the ratio measurement approach and assays were implemented in our laboratory after a request from members of the Australian rendering industry to develop a relative quantification method for rendered meat materials. Cow, pig, sheep, chicken and goat were identified as their species of highest priority, so the goat assay, published by Laube et al. [24], was implemented as it met the same assay design criteria for ratio measurements. To support the Australian pet food industry, horse, donkey, crocodile, turkey, duck, camel and kangaroo species were also added. Although Köppel et al. published a horse assay [19] that is widely used in Europe [25], this also amplified donkey DNA. With populations of feral horses and donkeys in Australia, this cross-reactivity could lead to ambiguity if an investigation were required. A new assay with greater species specificity was designed to discriminate between horse and donkey DNA. No suitable kangaroo, crocodile, duck or donkey assays could be found in the literature, so these were designed in-house. A camel assay was included following incidents of dog fatalities in 2019 due to consuming Australian camel meat containing Indospicine [26], resulting in camel being excluded from pet food in Australia. Lastly, to allow ratio quantification of cheese products, a water buffalo assay was included to account for all sources of dairy.

The animal species ratio method was intended to be used for routine testing of food, feed and MBM. One challenge that arises from the use of species-specific assays is whether there could be significant amounts of animal material that is not detected. Reference assays have been developed for this purpose, such as the mammalia family assay [27], universal 18S rRNA assay [13] and the myostatin reference assay [28]. The myostatin assay, targeting a single copy nuclear gene, was later modified with the introduction of adenine/guanine (A/G) degeneracy (wobble) in both the forward primer and probe to improve amplification efficiencies for poultry DNA [24]. In 2016, two further modifications to this assay were published [4] using alternative reverse primers and a probe that created amplicons of 20 or 27 bp shorter than the original 97 bp amplicon, referred to as MY77 and MY70, respectively. The assay modifications meant the amplicon lengths more closely matched those of two deer assays published in earlier work [4,29,30,31], reducing possible amplicon length bias seen when analysing heat-treated products. The shorter the DNA sequence being targeted, the more copies will remain intact after food processing that causes DNA fragmentation. Using assays with amplicons of different lengths can cause a ratio measurement bias favouring the shorter amplicons. The MY77 assay was selected for the ratio measurements in this work, but with further modifications in order to amplify marsupial and reptile DNA. The 1:1 ratios of MY77 targets with each of the new species assay targets were confirmed.

Routine testing of food and feed samples poses a range of challenges, as there are many factors that impact the quantity and quality of meat DNA that can be extracted for PCR analysis. These include food processing and purification steps that can either damage or remove DNA, or simple dilution effects with non-meat ingredients. Both processing and dilution can have a significant impact on the LOD of the measurement method. To support these challenges, the MY77 assay can be used for multiple quality control purposes in a routine food-testing context, including monitoring DNA extraction yield and purity, identification of the presence of meat species not detected by the present suite of species assays and potentially estimating sample-specific LODs based on the copy number concentration of DNA from each sample.

An in-house-designed synthetic DNA reference material (RM) was developed to simplify the commercial testing positive template control requirements. The availability of standardised assays and measurement approaches, supported by appropriate RMs, improves the reliability, comparability and transparency of food testing that increase confidence in the identity of food products. This ratio measurement approach has been demonstrated for a range of food and feed matrices, including MBM, fresh and cooked meat, cheese, canned wet pet food and kibble.

## 2. Materials and Methods

### 2.1. DNA, Meat and MBM Samples

Samples of fresh beef, pork, lamb, goat and chicken meat (muscle) were purchased from a local, reputable butcher who sourced produce directly from the farmers, with beef, pork and lamb samples cut directly from the carcass. Horse, donkey, camel, goose, quail, red deer, bison, alligator and alpaca DNA reference materials were purchased from the US company Zyagen, supplied by Banksia Scientific (Brisbane, QLD, Australia). Crocodile, kangaroo, turkey, duck and water buffalo DNA were extracted from fresh meat purchased from the supermarket. Single species (beef, pork, chicken, lamb and goat) rendered materials were kindly provided by members of the ARA: beef MBM and beef bonemeal were provided by Wingham Beef Exports (Wingham, NSW, Australia); pork MBM was provided by Primo Australia (Chullora, NSW, Australia); and chicken MBM, feather meal, lamb MBM and goat MBM were provided by Craig Mostyn (Fremantle, WA, Australia).

### 2.2. DNA Extraction

Where required, DNA was extracted using a modified version of the Promega Wizard^®^ Magnetic DNA Purification System for Food kit, using 0.1–0.2 g of sample. The rendered material was already in a powdered form; the fresh meat samples were finely chopped using a single-use, sterile scalpel blade. Samples were resuspended in Lysis Buffer A and RNase, according to the kit instructions, with the addition of 80 µL of proteinase K (Thermofisher Scientific, Waltham, MA, USA) prior to the 1 h incubation at 50 °C in an end-over-end mixer (Benchmark Roto-therm miniPlus, Sayreville, NJ, USA). Lysis Buffer B and Protein Precipitation solutions were added according to the kit instructions, followed by a 10 min centrifugation at 10,000 rpm (Eppendorf centrifuge 5810 R). DNA was extracted from the clarified supernatant using the magnetic beads from the extraction kit and a KingFisher Duo Prime (ThermoFisher Scientific). DNA was then eluted in 130 µL TE_0.1_ (10 mM Tris and 0.1 mM EDTA, pH 8.0). DNA concentrations (ng/µL) were initially estimated using UV spectrophotometry, then accurately measured (copy number/µL) using dPCR. Bio-Rad (Hercules, CA, USA) recommends digesting very high molecular weight genomic DNA prior to dPCR to reduce viscosity, so ~1 µg samples of DNA extracted from fresh meat were digested with *Sac*I in 1× CutSmart buffer (New England BioLabs, Ipswich, MA, USA) for 3 h at 37 °C and diluted 1/10th in TE_0.1_ prior to dPCR. None of the PCR amplicons targeted in this work contained *Sac*I restriction sites. The presence of PCR inhibitors co-eluting from the matrices was monitored using a 1:1 mix of extracted DNA and synthetic DNA RM to confirm there was no reduction in the fluorescence amplitude of the RM-positive droplets.

### 2.3. Droplet Digital PCR

Each droplet dPCR mix contained 12.5 µL 2xddPCR Supermix for probes without dUTP (Bio-Rad, catalogue number 1863024), 1 µL of primer/probe premix (final concentration of both primers: 900 nM, final concentration of Taqman^®^ probe: 250 nM) synthesised by Sigma (St Louis, MI, USA), 8 µL either fragmented or restriction digested DNA and nuclease-free water to make up a total volume of 25 µL. Reaction mixes were transferred to 96-well semi-skirted plates (Bio-Rad, catalogue number 12001925), sealed with a foil heat-seal and centrifuged for 30 s before being placed in the AutoDG (Bio-Rad), for droplet generation. The destination plate containing the droplets was then heat-sealed with foil and placed in a C1000 deep-well thermocycler (Bio-Rad) for temperature cycling as follows: 95 °C enzyme activation for 10 min; 40 cycles of 96 °C for 30 s and 60 °C for 60 s; 10-min hold at 98 °C, with a temperature ramp rate of 2.5 °/s. Where assays were run in duplex, the following assay primers and probes were pre-mixed: cow(F)/pig(H), sheep(F)/chicken(H), water buffalo(F)/goat(H), horse(F)/kangaroo(H), horse(F)/donkey(H), turkey(F)/duck(H), camel(F)/crocodile(H). The letter in brackets indicates the fluorophore attached to the probe: F = FAM and H = HEX. For dairy analysis, the following assay pairs were used: sheep(F)/beef(H) and water buffalo(F)/goat(H). All assay primer and probe details are listed in Table 1.

In the primer/probe sequences, the letter R is used to represent adenine/guanine (A/G) degeneracy and the letter Y is used to represent thymine/cytosine (T/C) degeneracy.

### 2.4. Preparing DNA Mixtures

Stocks of digested cow, pig, sheep, chicken, horse, goat and kangaroo DNA were diluted in TE_0.1_ to approximately 1000 copies/µL and the final concentrations were confirmed using dPCR. These DNA samples were then further diluted gravimetrically to 1/10th and 1/100th, with masses recorded using a 5-figure XP205 balance (Mettler Toledo, Greifensee, Switzerland). Six DNA mixtures were prepared volumetrically, using the dilution of DNA that allowed pipetting of volumes ranging from 10 to 95 µL.

### 2.5. Synthetic DNA RM

A DNA RM was designed, containing the individual amplicon sequences for cow, pig, sheep, chicken, EU horse, in-house horse, goat, water buffalo, kangaroo and myostatin assays in a 1:1 ratio and separated by *BamH*I restriction sites. This sequence was produced as a synthetic G-Block by IDT (Coralville, IA, USA) and the full-length construct was PCR amplified in-house using the KAPPA HiFi HotStart ReadyMix PCR Kit (Roche, Basel, Switzerland), purified by Ion Exchange HPLC [33] and desalted using magnetic AMPure^®^ XP beads (Beckman Coulter, Indianapolis, IN, USA) on a KingFisher Duo Prime (ThermoFisher Scientific) using the manufacturer’s instructions. The individual assay targets were separated by *BamH*I restriction digestion prior to quantification by dPCR. Separation of the different target templates in the RM allows them to partition independently, such as in a real sample containing genomic DNA contributed by multiple species, rather than partitioning together which would result in the majority of the positive partitions in a duplex reaction being positive for both targets. The purified DNA was then diluted to ~15 copies per µL to be used as a positive template control.

## 3. Results

### 3.1. Seven Novel Species-Specific Assays

**Figure 1:**
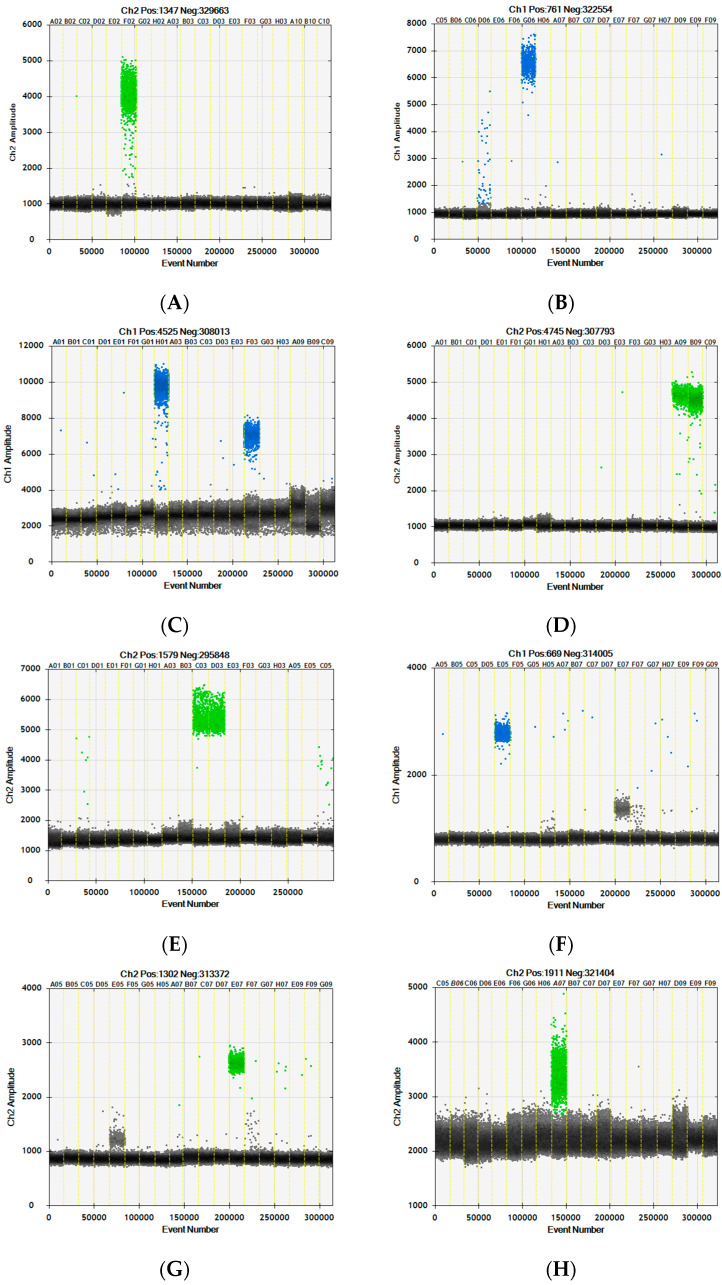
dPCR analysis of novel species assays. (**A**) Kangaroo assay. (**B**) Water Buffalo assay. (**C**) Camel/alpaca assay. (**D**) Crocodile/alligator assay. (**E**) Duck/goose assay. (**F**) Horse assay. (**G**) Donkey assay. (**H**) Published goat assay [24]. One-dimensional scatter graphs are generated by the Bio-Rad QuantaSoft^®^ software (version 1.7.4.0917), with each vertical column capturing data from an individual well. DNA samples analysed: cow, pig, chicken, sheep, horse, kangaroo, water buffalo, camel, goat, turkey, duck, goose, donkey, alpaca, red deer, bison, crocodile, alligator and quail. Droplets coloured blue or green demonstrate positive PCR amplification using FAM or HEX-labelled probes, respectively. Black droplets are PCR-negative. The dPCR assays were run as duplexes: water buffalo(FAM)/goat(HEX), camel(FAM)/crocodile(HEX), turkey(FAM)/duck(HEX), horse(FAM)/kangaroo(HEX), horse(FAM)/donkey(HEX). The horse results shown in F were duplexed with the donkey assay.

Novel species-specific assays for kangaroo, water buffalo, camel, crocodile, duck, horse and donkey were developed using alignments of gene sequences derived from GenBank reference genomes, targeting regions unique to each species. The assay criteria included genes present in a single copy per haploid genome, amplicon length of ~80–110 bp and annealing temperatures of ~60 °C. Selected assays were tested on a panel of DNA samples from meats used for human or pet consumption, to check for species specificity (Figure 1).

The kangaroo assay (Figure 1A) shows good species specificity, with no amplification using any other meat species included in this panel. The water buffalo assay (Figure 1B) produces some amplification when using sheep DNA due to sequence similarity in the assay region, but can be easily differentiated by the fluorescence amplitude of the positive populations in the y-axis. The very weak signal from sheep DNA is removed by increasing the PCR annealing temperature to 61 °C, but this change had a negative impact on some other species assays, particularly the goat assay (Figure 1H). For routine testing, it is highly preferable to have all assays amplified using the same PCR cycling program. The goat assay has lower amplification efficiency when using 60 °C annealing compared to the other species assays, shown by the small separation between the positive and negative droplet populations when using goat DNA. This is possibly due to the string of nine adenine ‘A’ nucleotides in the reverse primer (see Table 1). Attempts to redesign this primer whilst maintaining species specificity have not improved the assay performance. The camel assay (Figure 1C) also detects alpaca, as expected, but the positive droplets have a lower fluorescence amplitude when using alpaca DNA due to a single base mismatch within the probe binding region. This assay is also predicted to detect llama DNA, based on the prolactin gene sequence line-ups. The crocodile assay (Figure 1D) also detects alligator DNA, as expected. The duck/goose assay shows a weak signal from chicken and quail (Figure 1E). As with the water buffalo assay, this is eliminated by increasing the PCR annealing temperature to 61 °C.

**Figure 2:**
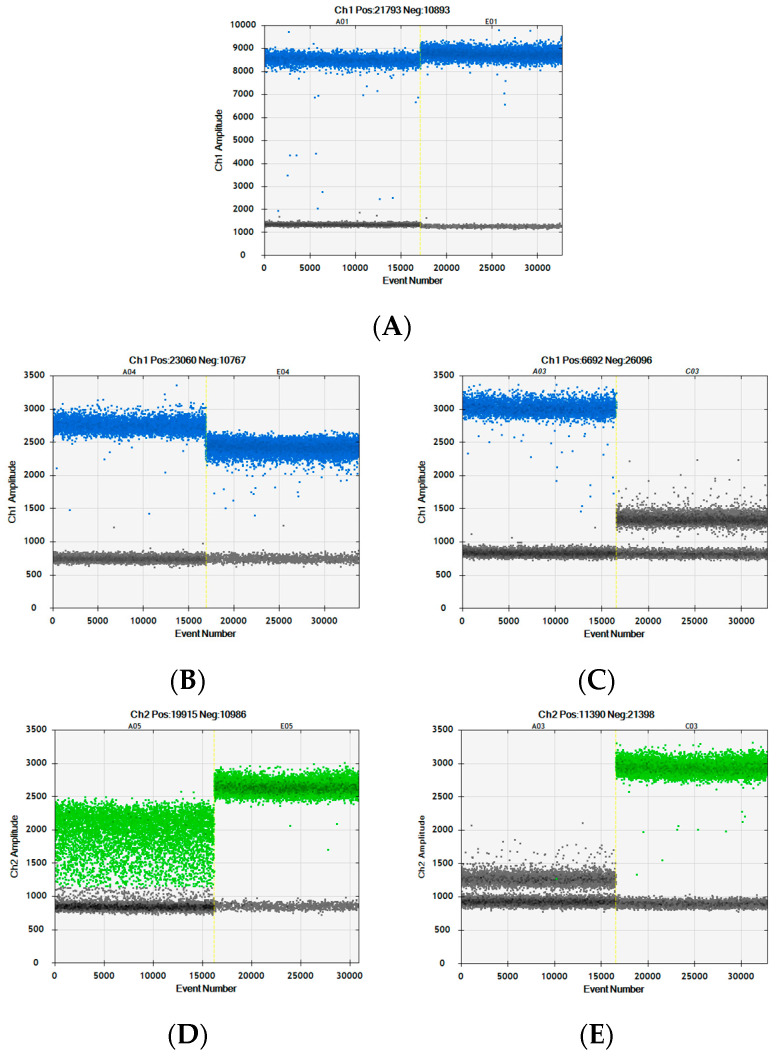
dPCR analysis of horse and donkey assays run in simplex and duplex. One-dimensional scatter graphs showing analysis of horse DNA (left well) and donkey DNA (right well). (**A**) European horse assay (simplex). (**B**) Novel horse assay (simplex). (**C**) Novel horse assay (duplexed with donkey assay). (**D**) Novel donkey assay (simplex). (**E**) Novel donkey assay (duplexed horse with assay).

The new horse assay (Figure 1F) was designed with the intention to be able to differentiate between horse and donkey DNA, since the European horse assay detects both with equal efficiency (Figure 2A). As horse and donkey genome sequences are extremely similar, it was challenging to identify DNA sequences suitable for assay designs that would differentiate between the two. Out of those trialed, the assay presented here produced the best specificity, though only when run as a duplex with the donkey assay. Likewise, the donkey assay (Figure 1G) works best when paired with the horse assay. Neither the horse nor donkey assays produce the desired species specificity when run as simplex reactions (Figure 2B,D). The donkey and horse assays target the same DNA sequence, with the 3′ ends of both primer pairs binding to bases that differ between horse and donkey sequences and with the probes differing by a single nucleotide. When run as a duplex, the competition for binding between the perfectly matched and mismatched primers and probes significantly improves assay specificity, allowing DNA from horse or donkey to be clearly distinguished (Figure 2C,E). Positive template control sequences are used for direct comparison, to ensure droplet populations are defined correctly.

### 3.2. Modifications to the Myostatin Assay

**Figure 3:**
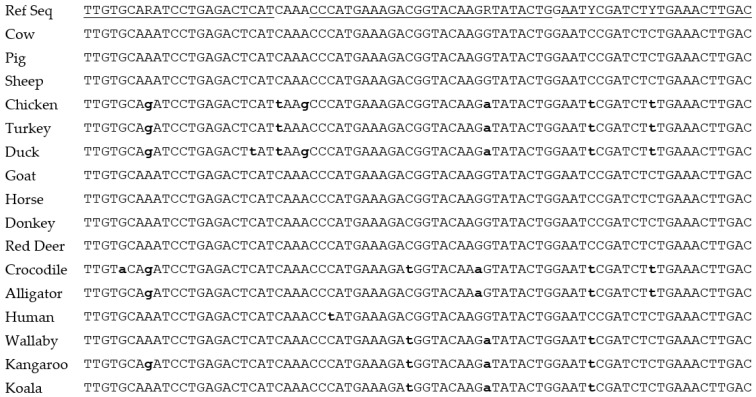
Sequence line-up of the myostatin amplicon targeted by the MY77 PCR assay [4]. Underlined sequences in the reference sequence indicate the binding positions for the published MY77 PCR assay and include the wobbles introduced to match the single nucleotide variants [4,24]. The nucleotides highlighted in bold and lowercase are those that deviate from the reference sequence.

An “all four-limbed species” positive control assay was required to confirm whether the meat detected using species-specific assays represented all the meat present in the sample. This also needed to be a single copy nuclear target for relative quantification purposes. The published myostatin assay [28] was ideal for this purpose and had already gone through two rounds of modifications, to shorten the amplicon and the introduction of a wobble base in position 21 of the probe to improve the detection of poultry DNA [4,24]. Coincidentally, this wobble also improved the detection of marsupial DNA (see Figure 3). During in-house assay verification of this modified MY77 assay, the annealing temperature was assessed on the Bio-Rad C1000 end-point PCR platform which was used for dPCR. The optimal temperature was found to be ~57 °C when using cow and pig DNA, with a significant drop in performance when using 60 °C annealing. The optimal temperature was even lower for species with an A or T in the wobble positions. To increase the annealing temperature for all species, the theoretical melt temperature (Tm) of the primers was assessed using the IDT OligoAnalyzer™ Tool software and the reverse primer appeared to have the lower Tm of the pair. From the alignments of representative myostatin sequences (Figure 3), it was confirmed that a 2-base extension at the 3′ end of the reverse primer would increase the annealing temperature whilst having no impact on assay specificity.

**Figure 4:**
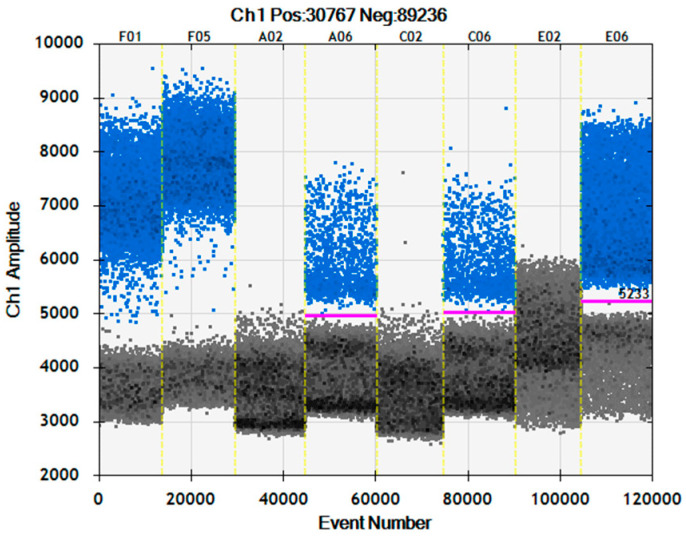
Comparison of performance between the published MY77 assay and a modified version of this assay using a novel reverse primer and probe to expand species suitability. DNA samples: chicken (wells 1–2), kangaroo (wells 3–4), crocodile (wells 5–6) and alligator (wells 7–8). For each species, the first well shows the MY77 assay published by Druml et al. [4] (primer and probe sequences underlined in Figure 3); the second well shows the MY77 assay using a longer reverse primer (MY77R_251895) and the new probe (MY97P_256895F_YR), including the second wobble at position 12. The pink lines indicate where the boundaries between positive and negative populations were added manually.

Since kangaroo and crocodile meat are used for both human and animal consumption, for the Australian testing market it was important for these species to be detected by the all-meat positive control. Marsupial and reptile myostatin sequences were added to the myostatin amplicon sequence line-up (Figure 3). This allowed the identification of a second mismatch at position 12 within the probe binding region for the three marsupial sequences, wallaby, koala and grey kangaroo. The same substitution is found in the crocodile sequence, along with a third mismatch at position 20 for both crocodile and alligator. The probe was redesigned to include the position 12 wobble (C/T). As each additional wobble can have a negative effect on probe performance by reducing the pool of probes with 100% match for individual sequences, the third wobble was not included. The performance of the previous and novel MY77 assays were compared (Figure 4).

The chicken dPCRs produce a higher fluorescence amplitude when using the longer reverse primer (MY77R_251895) due to an increase in the assay annealing temperature. The kangaroo, crocodile and alligator dPCRs did not amplify using the original version of the MY77 assay but were improved significantly using the modified assay. Although the separation of the positive and negative populations is not as big for the kangaroo, crocodile and alligator DNA when compared to the chicken DNA, there is still clean separation to allow the two populations to be distinguished.

In a routine testing context, the myostatin assay can serve multiple purposes: as a positive extraction control assay for each sample being analysed; as an inhibition control when sample DNA is mixed 1:1 with an independent positive control DNA sample; and as an indicator to show that all species of meat present in the sample have been accounted for by the species-specific assays used, or whether there is other, undetected meat present. As the animal species and myostatin assays are all present in a single copy per haploid genome, the sum of the copy numbers from each individual species detected should match the copy numbers of the myostatin target when all species in the sample have been detected, within the uncertainty of the measurement method.

### 3.3. Confirmation of Copy Number Ratio between Myostatin and Species-Specific Assay Targets

**Figure 5:**
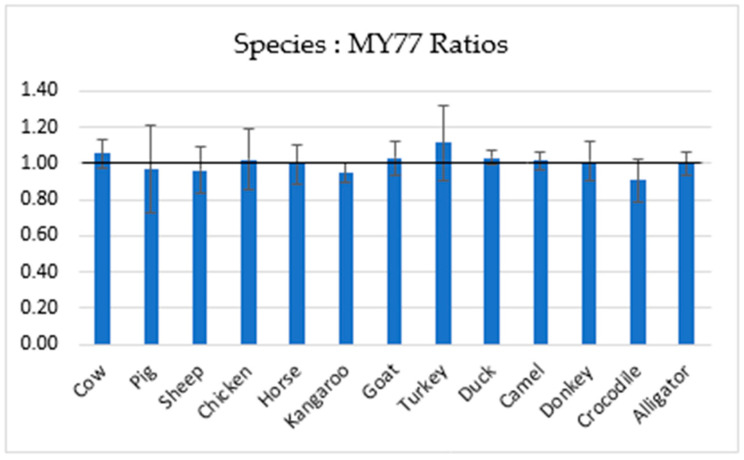
Copy number concentration ratios for each of the animal species assays relative to myostatin. Ratios were calculated as the copy number concentration from the species-specific assay divided by the copy number concentration from the MY77 assay. The error bars show two standard deviations (95% coverage) from four replicate ratio measurements.

This measurement approach was expected to produce a 1:1 copy number ratio between the species-specific and myostatin assay targets, based on whole genome sequence analysis, and this was confirmed from the dPCR measurements of DNA extracted from fresh meat (Figure 5).

### 3.4. Ratio Measurements from Meat Mixtures

**Figure 6:**
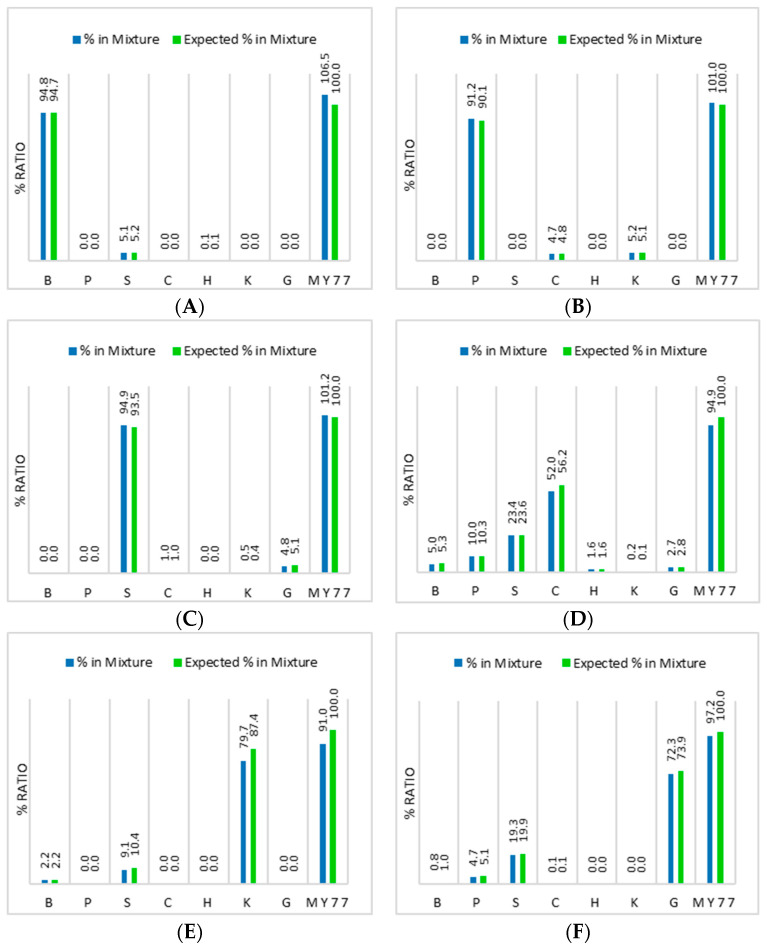
Measured versus expected DNA copy number ratios (%) for the DNA mixtures prepared, as shown in Table 2, using seven of the animal species-specific assays. (**A**) Mixture 1. (**B**) Mixture 2. (**C**) Mixture 3. (**D**) Mixture 4. (**E**) Mixture 5. (**F**) Mixture 6. Measured (blue) and calculated (green) copy number ratios for mixtures of extracted DNA. B = beef, P = pork, S = sheep, C = chicken, H = horse, K = kangaroo, G = goat. The expected ratios for the individual species were calculated using the sum of species-specific copies as the denominator (Equation (1) shows the beef ratio calculation). The measured ratios for each of the species are the average copy number concentrations of duplicate dPCRs for each assay, as a proportion of the total dPCR copy number concentrations measured for that sample. The expected value for myostatin (100%) was calculated from the sum of species-specific copies added to the mixture, using the pre-measured DNA copy number concentrations and DNA volumes. The measured ratio for myostatin was calculated from the MY77 copy number concentrations relative to its expected copy number value.

To assess the performance of the dPCR approach to measure accurate ratios of the different meat species, digested and quantified DNA from seven species (cow, pig, sheep, horse, chicken, kangaroo and goat) were mixed in different ratios (Table 2) and then quantified using dPCR (Figure 6).

(1) $$Expected\;ratios\;B\;\%=\left(\frac{B}{B+P+S+C+H+K+G}\right)\times 100$$

The results in Figure 6 show that duplex dPCR results can produce accurate measurement ratios down to 0.1% when using mixtures of high-quality and purity genomic DNA and that the measured myostatin copy number concentration is within 10% of the sum of the individual species targets.

### 3.5. Synthetic DNA Reference Material

**Figure 7:**
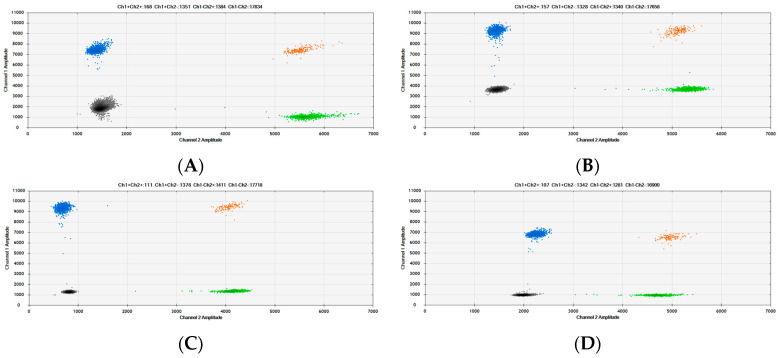
Two-dimensional scatter graphs generated by the Bio-Rad QuantaSoft^®^ software (version 1.7.4.0917) of the synthetic DNA animal species reference material, RM1, using duplex dPCR assays. The animal species assays are analysed in pairs using four primers and two probes: (**A**) Cow/pig duplex assays. (**B**) Sheep/chicken duplex assays. (**C**) Horse/kangaroo duplex assays. (**D**) Water buffalo/goat duplex assays. The FAM-labelled probes (cow, sheep, horse and water buffalo assays) are analysed in Channel 1 and viewed on the y-axis. The HEX-labelled probes (pig, chicken, kangaroo and goat assays) are analysed in Channel 2 and viewed on the x-axis. In each scatter graph, black populations represent negative droplets that contain neither of the species targets, the blue populations contain targets for the FAM-labelled assay only, the green populations contain targets for the HEX-labelled assay only, the orange populations contain targets for both assays. When quantifying the number of positive droplets, the FAM assay is the sum of the blue and orange populations and the HEX assay is the sum of the green and orange populations.

Digital PCR can produce quantitative data in the absence of a calibrant, but it still requires a positive control to validate assays, to confirm that the PCRs are set up properly, and to correctly define the identity of populations, particularly when running multiplex reactions. This reduces the risk of false-negative reporting. A mixture of extracted DNA from each of the target species was originally prepared and analysed in parallel with commercial samples. For some of the assays, this has been replaced by a synthetic DNA RM that includes assay targets for the cow, pig, sheep, chicken, horse, kangaroo, water buffalo, goat and myostatin PCRs in a 1:1 ratio (Figure 7).

### 3.6. Digital PCR Performance Is Impacted by Processing of the Meat Samples

**Figure 8:**
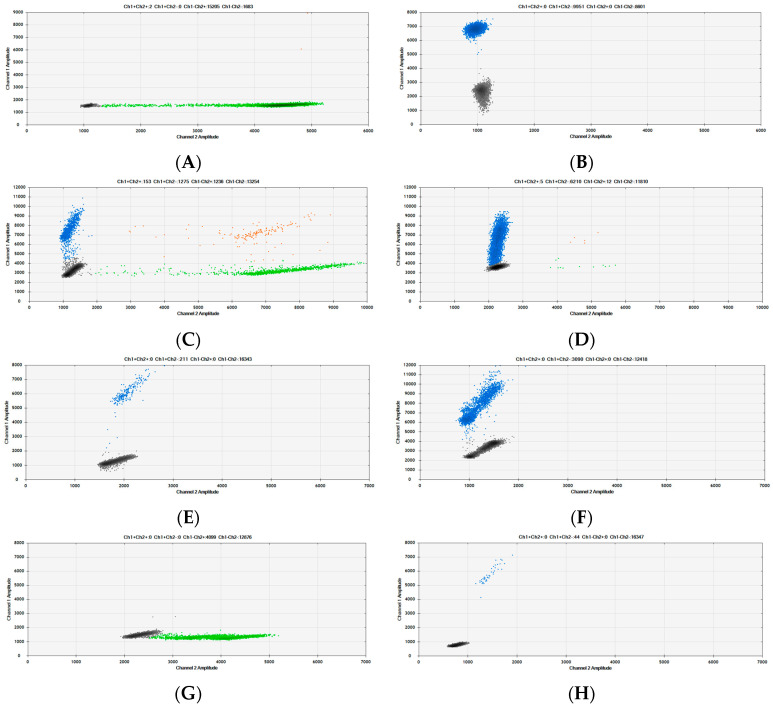
Examples of food, pet food and MBM analysed using duplex dPCR. (**A**) Kangaroo meat dog food roll. (**B**) Teriyaki beef strips. (**C**) Beef and pork MBM. (**D**) Rendered ovine MBM. (**E**) Cow’s milk camembert cheese. (**F**) Sheep’s milk feta. (**G**) Goat’s milk hard cheese. (**H**) Water buffalo blue cheese.

The data presented above demonstrate the performance of the dPCR assays and ratio measurement approach using either a synthetic DNA RM or animal DNA that has been extracted from fresh meat and restriction digested. This represents best-case scenarios using the highest quality DNA. When analysing food and feed samples, the quality and quantity of DNA can be compromised, which will impact ratio accuracy and LOD. Cooking, canning and/or rendering processes all result in DNA fragmentation, with DNA extracted from autoclaved meat being 99% degraded with respect to fragment size [6]. DNA fragmentation causes droplet “rain”, or positive droplets with lower levels of fluorescence. As fragmentation increases, the positive and negative droplet populations can merge (Figure 8A,D), leading to an underestimation of the target copy number.

Food processing that either removes or damages DNA will impact the LOD, but the MY77 concentration measurement could potentially be used as a surrogate to estimate the sample-specific LOD, as it captures both DNA yield and integrity factors. Although this method reports relative quantification (%), the ratios are calculated from absolute copy numbers of the DNA targets that could be amplified. Using the copy number concentration per well of amplifiable myostatin targets and a reporting cut-off of 10 copies, the LOD for the goat’s cheese (Figure 8G) LOD would be ~0.7% (1485 copies of MY77) but the LOD for the water buffalo blue cheese (Figure 8H) would be ~15% (65 copies of MY77). These estimated LODs were calculated using the average copies per reaction from duplicate dPCR measurements of duplicate DNA extractions to demonstrate the concept of a sliding scale for the LOD based on the amount of amplifiable DNA available.

## 4. Discussion

An animal species ratio dPCR method was adopted based on published species-specific PCR assays that target genes present in single copies per haploid genome, allowing cell ratios to be measured, independent of tissue type [18,21,24]. This method has been validated for analysis of rendered MBM to support the meat rendering and pet food industries. In this study, the range of target species has been expanded to include kangaroo, horse, donkey, duck, camel, water buffalo and crocodile to accommodate the species of meats likely to be included in Australian food, pet food and feed, or excluded from Australian pet food, as in the case of horse and camel meat. Although the focus of this work was to support the Australian pet food and meat rendering industries, the new assays are equally suited to food analysis, allowing DNA copy number ratio measurements to be reported, and providing more information than qualitative (presence/absence) results.

For reliable routine testing, particularly when methods are accredited against quality standards [34], positive and negative controls are required to monitor the success of each step. In the testing context presented here, positive and negative controls are required for both DNA extraction and PCR amplification. A PCR assay that can be used to detect all meat species is particularly useful to confirm that extracted DNA is of a suitable quantity, quality and purity for amplification. The published myostatin assay [4,24] was ideal for this role and has been further modified in this work to extend the range of animal species it can detect to encompass marsupials and reptiles. The 1:1 ratio of myostatin to species-specific PCR targets was confirmed for all the new assays using DNA extracted from fresh meat or purchased RM DNA. Very accurate ratio measurements were demonstrated using mixtures of high-quality and purity DNA, with excellent correlation between expected and measured DNA ratios. As the myostatin assay detects DNA from all four-limbed species in a sample, it can also be used to indicate whether a meat species not included in the suite of species-specific assays is present, when copy numbers of myostatin are significantly greater than total copies from the species-specific assays, potentially indicating adulteration or contamination.

For ratio measurements, there was a deliberate decision to target DNA sequences present in a single copy to improve ratio accuracy, accepting the consequence that it is a less sensitive method when compared to mitochondrial PCR assays. Mitochondrial assays are still preferable when extremely sensitive presence/absence results are required. When analysing DNA from fresh meat samples, the LOD of this method can go down to 0.1% with high levels of accuracy, which would be fit-for-purpose for most food adulteration requirements. However, the LOD is heavily influenced by the sample itself. DNA extracted from processed food and feed can produce a “rain” effect, with droplets of an intermediate fluorescence or positive and negative droplet populations merging. This rain can be caused by significant PCR inhibition from the matrix or by fragmentation of the DNA. DNA purity can be improved but not the fragmentation. For food or feed including non-meat ingredients, the extracted meat DNA will be diluted by non-target DNA and as the overall proportion of meat decreases, so will the copy number concentration of meat DNA, with the measurement method becoming progressively less sensitive. Processing that results in DNA degradation or removal also impacts the LOD. Cheese varieties vary considerably in the amount of extractable DNA, with LODs between ~1 and 15% depending on DNA yield. Gelatine contains only trace amounts of DNA and is not suitable for analysis with the PCR assays described here. Acid hydrolysis, used to reduce proteins to their composite amino acids, will completely hydrolyse DNA, making it unavailable for amplification by PCR. Where ingredients have undergone different processing prior to combining, this will impact species ratio accuracy. However, processing should not significantly alter species ratios when all ingredients are treated together, provided the PCR amplicons are of similar lengths [4]. The LOD is therefore a product of both the measurement method design and factors associated with the individual samples. Since the MY77 copy number concentration reflects both DNA yield and integrity, it has the potential to be used as a surrogate, producing a rough estimate of the sample-specific LOD for each animal species’ result.

The dPCR method was adopted due to several benefits over the earlier qPCR approach. Digital PCR is a counting method: provided there is clear differentiation between positive and negative droplets or partitions, low levels of PCR inhibitors can be present without compromising quantitative accuracy, unlike qPCR, which is affected by even low levels of inhibitor, a factor contributing to a lower measurement uncertainty when compared to qPCR [21,22]. Digital PCR has a dynamic range of multiple orders of magnitude [23], making it suitable for food fraud applications, and more importantly, it does not require a calibrant in order to produce quantitative results. There is still a requirement for a positive template control for each PCR assay, particularly when running multiplex reactions to define each population correctly. A synthetic DNA RM has been developed in this work to be used as a positive template control. It includes PCR targets for the cow/bison, pig, sheep, chicken, goat, kangaroo, horse, EU horse, water buffalo and myostatin assays in a 1:1 ratio. This RM is commercially available, with a second synthetic RM in the pipeline that will include PCR targets for donkey, turkey, duck, camel/alpaca and crocodile/alligator assays. The use of standardised RMs and measurement approaches improves the reliability of quantitative results, leading to comparability between testing service providers and greater confidence in testing results.

## Figures and Tables

**Table 1:** Primers and probes used in this study.

Species	Name	Primer/Probe Sequence	Length
^1^ Beef Assay, beta-actin gene	Rd 1 F	GTAGGTGCACAGTACGTTCTGAAG	96 bp
	Rd 1 R	GGCCAGACTGGGCACATG	
	Bos-ActiB_FAM	FAM-CGGCACACTCGGCTGTGTTCCTTGC-BHQ1	
^2^ Pig Assay, beta-actin gene	Sus_ACTB-F	GGAGTGTGTATCCCGTAGGTG	103 bp
	Sus_ACTB-R	CTGGGGACATGCAGAGAGTG	
	Sus1_TMP	HEX-TCTGACGTGACTCCCCGACCTGG-BHQ1	
^2^ Sheep Assay, prolactin receptor	OA-PRLR-F	CCAACATGCTTTTAAACCCTCAA	88 bp
	OA-PRLR-R	GGAACTGTAGCCTTCTGACTCG	
	OA-PRLR-FAM	FAM-TGCCTTTCCTTCCCCGCCAGTCTC-BHQ1	
^1^ Chicken Assay, growth factor gene	Gallus1 F	CAGCTGGCCTGCCGG	76 bp
	Gallus1 R	CCCAGTGGAATGTGGTATTCA	
	Gallus1 TMP	HEX-TCTGCCACTCCTCTGCACCCAGT-BHQ1	
^6^ Water Buffalo Assay, beta-actin gene	BubF_689776	GTGCACAATACGTTCTGAAGTG	110 bp
	BubR_688804	CCACAAGGGGCAGTCAA	
	BubP_686035F	FAM-ATCCCCAGCACACTTAGCTGTGTTCC-BHQ1	
^3^ Goat Assay, cyclic GMP phosphodiesterase	capraPDE-f	TACCCATCAAGCAGACTCTAGCA	96 bp
	capraPDE-r	ATATTTCAGCTAAGGAAAAAAAAAGAAG	
	capraPDE-probe	HEX-ATTTTTGTCGCATTCGCTTCATCTGT-BHQ1	
^2^ Horse Assay, growth hormone receptor	ec-ghr1-F	CCAACTTCATCATGGACAACGC	107 bp
	ec-ghr1-R	GTTAAAGCTTGGCTCGACACG	
	ec-ghr1-Cy5	FAM-AAGTGCATCCCCGTGGCCCCTCA-BHQ1	
^6^ Horse Assay, prolactin gene	EquF_638806	GCAGTTGACAGCCCCACTT	74 bp
	EquR_636141	TGCTGGTGTCAGATCTACTCT	
	EquP_632044F	FAM-GGGAGGCCACACTCTTGCACAAGAG -BHQ1	
^6^ Donkey Assay, prolactin gene	AsiF_841634	GCAGTTGACAGCCCCACTC	74 bp
	AsiR_848969	TGCTGGTGTCAGATCTACTCC	
	AsiP_844970H	HEX-GGAGGCCACGCTCTTGCACAGG-BHQ1	
^6^ Kangaroo Assay, omega globin gene	KanF_729413	GCGTTGGGCTAAACTAGGTT	91 bp
	KanR_728566	TCCTCTACCACATCCTCCAC	
	KanP_721842H	HEX-TGCGGGACCCTGGTCATGAGTGCTT-BHQ1	
^4^ Turkey Assay, prolactin receptor	MG-ProlR-F	CAAAGAAAGCAGGGAAAAGGA	83 bp
	MG-ProlR-R	TGCACTCTCGTTGTTAAAAAGGA	
	MG-ProlR-Cy5	FAM-CTGGGAAAGTTACTGTGTAGCCTCAGAACG-BHQ1	
^6^ Duck Assay, prolactin receptor	AnaF_407450	GAGATGTTCAAGAAAATAAAGCTGT	91 bp
	AnaR_408406	CTCTCACTGTTAGAAAGGAGTG	
	AnaP_403910H	HEX-TGGGAAACTCAGTGTGTAGCCTCAGAACGG-BHQ1	
^6^ Camel Assay, prolactin gene	CamF_309073	CAGTTGACAGCCCCGCTG	102 bp
	CamR_308908	TTAAGCAGGGTCGCTCTTGT	
	CamP_305227F	FAM-CACGCTGTTGCACAAGAGCAGATTTG-BHQ1	
^6^ Crocodile/Alligator Assay, insulin gene	CrocF_470384	TCTAGCCCCAGTGTCAGCTA	91 bp
	CrocR_471332	CCCTTTCACCACACACCAGA	
	CrocP_477150H	HEX-CCAGCGCCTGTGTGGCTCTCAC-BHQ1	
Myostatin 77 bp Assays	^3^ MYw-f	TTGTGCARATCCTGAGACTCAT	77 bp
	^5^ MY77 reverse	GTCAAGTTTCARAGATCGRATT	
	MY77R_251895	GTCAAGTTTCARAGATCGRATTCC	
	^3^ MYw-probe	FAM-CCCATGAAAGACGGTACAAGRTATACTG-BHQ1	
	MY97P_256895F_YR	FAM-CCCATGAAAGAYGGTACAAGRTATACTG-BHQ1	

^1^ [20]; ^2^ [19]; ^3^ [24]; ^4^ [32]; ^5^ [4]. ^6^ Here.

**Table 2:** Predicted copy ratios (% DNA, cp/cp) from mixtures prepared from purified DNA of known concentrations.

	Mixture 1	Mixture 2	Mixture 3	Mixture 4	Mixture 5	Mixture 6
Beef DNA	94.7%	-	-	5.3%	2.2%	1.0%
Pig DNA	-	90.1%	-	10.3%	-	5.1%
Sheep DNA	5.2%	-	93.5%	23.6%	10.4%	19.9%
Chicken DNA	-	4.8%	1.0%	56.2%	-	0.1%
Horse DNA	0.1%	-	-	1.6%	-	-
Kangaroo DNA	-	5.1%	0.4%	0.1%	87.4%	-
Goat DNA	-	-	5.1%	2.8%	-	73.9%

## Data Availability

The data presented in this study are available on request from the corresponding author.
